# PD-1 and CTLA-4 serve as major gatekeepers for effector and cytotoxic T-cell potentiation by limiting a CXCL9/10-CXCR3-IFNγ positive feedback loop

**DOI:** 10.3389/fimmu.2024.1452212

**Published:** 2024-10-15

**Authors:** Noor Abdala-Saleh, Jennie Lugassy, Akshatha Shivakumar-Kalvhati, Abeer Turky, Sari Abu Ras, Hila Razon, Nir Berger, Dana Bar-On, Yotam Bar-On, Tetsuya Taura, David Wilson, Nathan Karin

**Affiliations:** ^1^ Department of Immunology, Faculty of Medicine, Technion, Haifa, Israel; ^2^ Research and Development, Teva Pharmaceutical Industries, Ltd., Netanya, Israel; ^3^ Biologics Discovery, Teva Pharmaceutical Industries Ltd, Redwood City, CA, United States

**Keywords:** CXCL9, CXCL10, CXCR3, PD-1, ICI

## Abstract

CXCR3 is a chemokine receptor with three ligands: CXCL9, CXCL10 and CXCL11. We report that in addition to attracting CXCR3+ T cells to tumor sites a key role of CXCL9 and CXCL10 is in inducing a self-feeding feedback loop that accelerates effector/cytotoxic activities of both CD4+ and CD8+ T cells while downregulating immunoregulatory protein TIM3. CXCR3KO mice displayed a markedly reduced response to anti-PD-1 and anti-CTLA-4 therapy. Results from a panel of *in vivo* and ex vivo 3D tumor models imply that, beyond driving CD8+ T cells into T-cell exhaustion, a major role of PD-1 and CTLA-4 is in limiting the CXCR3-based self-feeding mechanism of T cell potentiation. This may explain why patients that are CXCL9/CXCL10^high^ tend to respond well to anti-PD-1 therapy, as opposed to patients that are CXCL9/CXCL10^low^. It also suggests a therapeutic role for CXCL9-Fc or CXCL10-Fc therapy; herein we demonstrate significant anti-tumor activity in multiple murine tumor models with such agents.

## Introduction

The most important breakthrough in cancer immunotherapy to date has been the development of immune checkpoint inhibitors (ICI), also referred as immune checkpoint blockers (ICB), initially with the pharmacological blockade of the interaction between cytotoxic T lymphocyte-associated antigen 4 (CTLA-4) and CD28, and soon followed by the blockade of programmed cell death 1 receptor (PD-1) with its ligand PD-L1 and PD-L2; progress with other ICIs has recently been reviewed by Sharma (1).

Immune checkpoint therapy (ICT) with ICI has successfully been extended to almost 20 cancer types, with PD-1 blockade showing the highest level of anti-tumor activity. Metastatic melanoma is the most responsive tumor type for ICT, with about 17% responding to anti-PD-1 antibody, about 27% responding to anti-CTLA-4 antibody, and about 55% responding to their combined therapy (2). Overall, about 10% of treated cancer patients respond to PD-1 blockade.

CXCR3 is a chemokine receptor with 3 ligands, CXCL9, CXCL10 and CXCL11. CXCL9 and CXCL10 bind a similar site on CXCR3, differing from that of CXCL11 (3). CXCR3 is primarily expressed on CD4+ and CD8+ T cells, and to some extent by certain dendritic cells (DC) (4), macrophages (5), NK cells (6–8), and epithelial cells (9). Within the CD4+ subset, CXCR3 is mostly highly expressed on effector T cells, and a small portion of FOXp3+ regulatory T cells (T_regs_). Thus, it is also associated with T_regs_ migration, including into tumors (10–12).

In numerous cancers, among them melanoma, non-small cell lung cancer (NSCLC), ovarian cancer, gastric cancer, and colorectal cancer, high CXCL9/CXCL10 levels indicate favorable prognosis, and low levels indicate poor prognosis (13–19). Moreover, melanoma patients who express low levels of CXCL9 or CXCL10 (either blood or tumor site) are poor responders to ICT, and those with high levels of CXCL9 or CXCL10 respond more favorably (18, 19). Likewise, NSCLC patients with high plasma levels of CXCL9 and CXCL10 displayed better responses to anti-PD-1 or anti-PD-L1 therapies (13). Specifically for CXCL9, Seitz et al. reported that CXCL9 inhibits tumor growth and drives anti-PD-L1 therapy in ovarian cancer (20) and that CXCL9 could be a potential biomarker of immune infiltration associated with favorable prognosis in estrogen receptor-negative breast cancer (21).

What may explain the association between CXCL9 and CXCL10 expression and cancer prognosis, as well as the response to ICT? Based on studies from different laboratories including ours, it was suggested that CXCL10, and possibly CXCL9, are not only associated with the migration of T cells, but also with directing their biological functions (22–27). We suggest that a combined function of targeted migration and directional polarization of CD4+ and CD8+ T cells account for the driver role of CXCR3 and its ligands in the immune rejection of tumors and responsiveness to ICT (28–30). Of these ligands, CXCL11 may differ from the other two by its ability to skew effector T cells into FOXp3-negative T regulatory-1-like cells (27).

The working hypothesis of the current manuscript is that the CXCR3-CXCL9/10 axis is a key driver of effector and cytotoxic CD4+ and CD8+ T cell function and that this axis maintains a self-feeding amplification loop in which the interaction of CXCL9 and CXCL10 with CXCR3 induces different subtypes of effector and cytotoxic CD4+ and CD8+ T cells with an IFNγ signature; since CXCL10 is IFNγ inducible (31, 32), high levels of IFNγ then further induces CXCL10 production, not only by T cells but also by cancer cells, thus reinforcing the loop. This positive feedback loop is likely to be regulated by CTLA-4 and PD-1 as key checkpoints. Here, using 3-D spheroids and CD8+ T cells we show that blockade of PD-1 or CTLA-4 further activates this loop. This effect may explain why melanoma and NSCLC patients that are CXCL9/10^low^ are poor responders to ICT, and perhaps that they could be potential candidates to combined therapies with ICT + CXCL9-Fc or CXCL10-Fc.

## Results

### Immune checkpoint inhibition is CXCR3 dependent

The response to anti-PD-1 and anti-CTLA-4 ICT in wild-type (WT) and CXCR3KO mice has been evaluated using the *Ret* transgenic mouse model, a skin malignant melanoma model that is characterized by the overexpression of the human *Ret* transgene in melanin-expressing cells (33). These transgenic mice spontaneously develop skin tumors with metastases to lymph nodes, lungs, liver, brain, and bone marrow (33). A low passage pre-line that was isolated from these mice (34) and then subjected to overexpression of mCherry (35), was used in the study. This serves as a reliable model for autotropic melanoma, with clear metastatic spread (34, 35).

**Figure 1:**
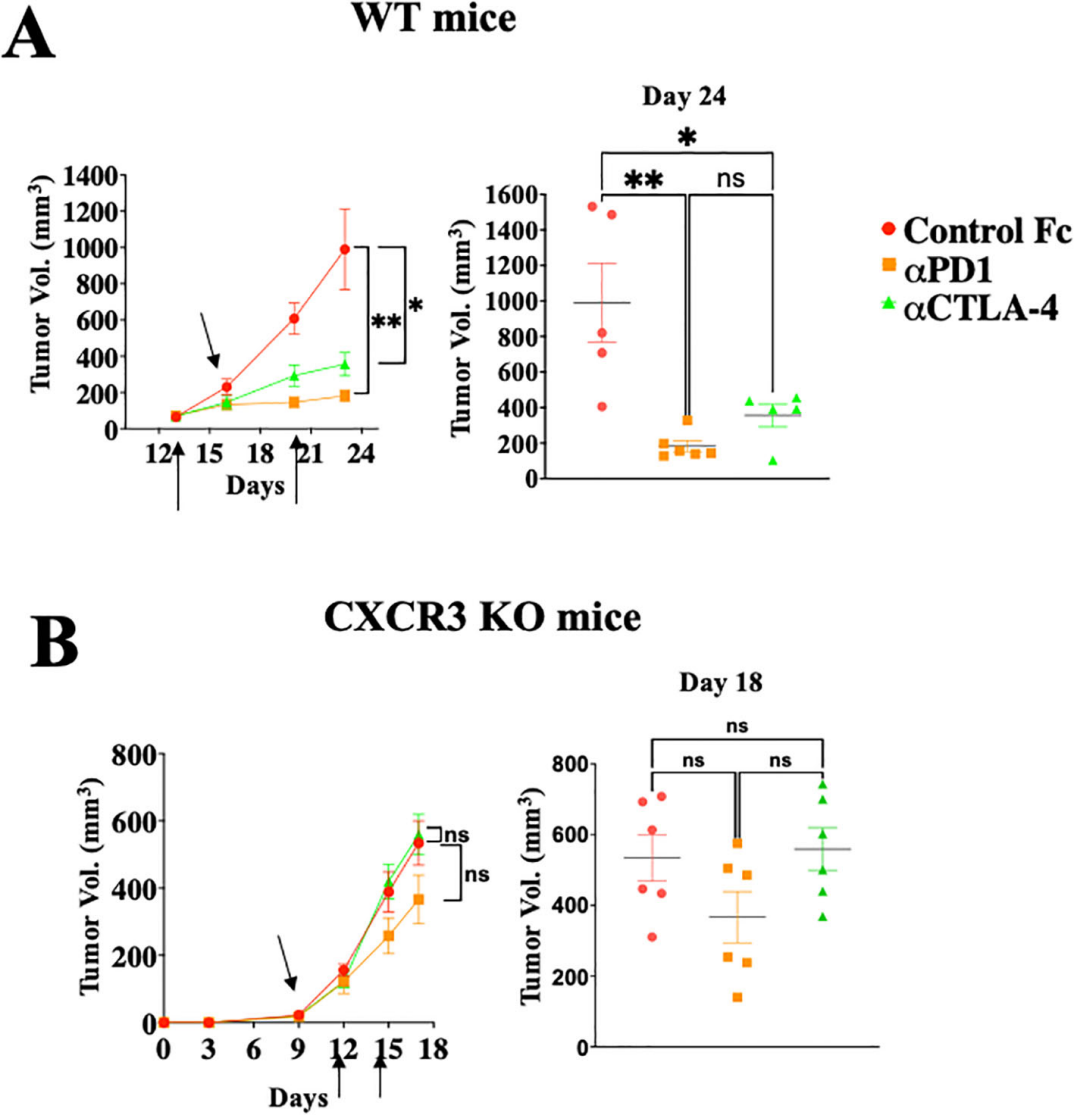
Immune checkpoint inhibition is CXCR3 dependent. WT **(A)** or CXCR3KO **(B)** mice were orthotopically injected with 0.4x10^6^ *ret* melanoma cells. When tumor size reached about 50 mm^3^, they were separated into three identical groups of six mice each. Each group was treated three times, in 3–4-day intervals with 100µg/mouse αPD-1 or αCTLA-4 or isotype-matched control IgG. Tumor volumes were recorded by an observer blinded to the experimental protocol. Results represent data of one of three independent experiments with similar observations. Significance was determined using a one-way analysis of variance (ANOVA) test using Tukey’s multiple comparisons test; *P≤ 0.05, **P≤ 0.01, ***P≤ 0.001 were considered significant. ns, not significant.

**Figures 1A, B** shows that WT mice engrafted with *Ret* melanoma display strong antitumor responses to blockade of either PD-1 or CTLA-4 (**Figure 1A**; P<0.05 for CTLA-4 and P<0.01 for PD-1 blockade), whereas these effects were much reduced in CXCR3 KO mice (**Figure 1B**). From the translational perspective, the findings that CXCL10l^high^/CXCL9^high^ patients display less severe forms of several types of cancer (13–19), and that melanoma patients who are CXCL10L^high^ and/or CXCL9^high^ tend to respond well to anti-PD-1 therapy (18, 19), suggests that the major role of the PD-1 and CTLA-4 axes is to regulate the CXCR3-CXCL9/CXCL10 interplay. This subject is further discussed below.

### CXCL9 and CXCL10 directly induce different subtypes of effector and cytotoxic CD4+ T cells and CD8+ T cells in a self-feeding loop and are likely to be regulated by anti-PD-1

**Figure 2:**
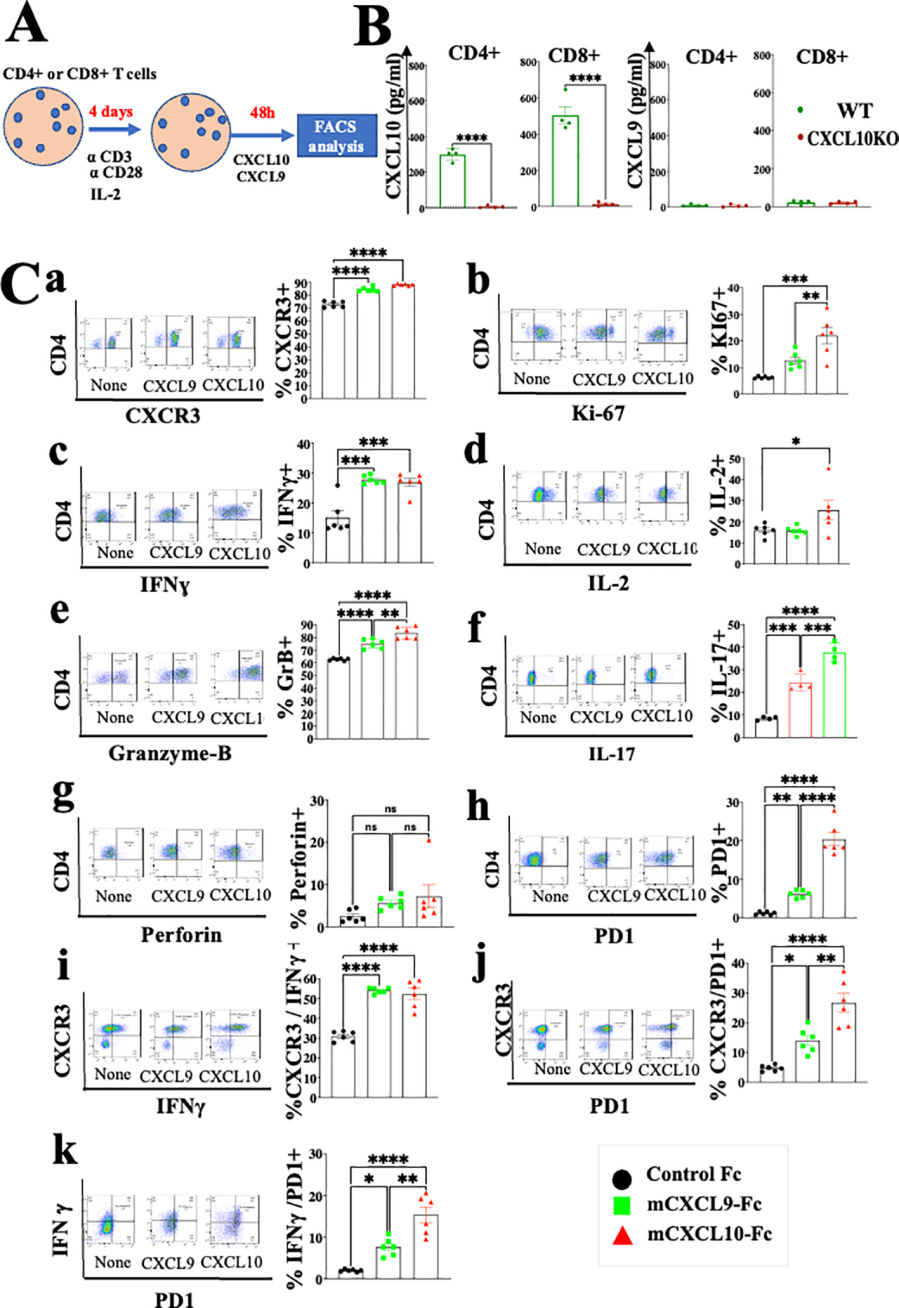
CXCL10 and CXCL9 drive the polarization of CD4+ T cells into IFNγ^high^ cytotoxic T cells and Th17^high^ CD4+ T cells while increasing their Ki-67 and IL-2 expression. **(A)** Spleenocytes were obtained from naïve mice (WT) or CXCL10 knockout (KO) mice (6 per group) and sorted into CD4+ cells using a MACS Easy-Sep magnetic separation kit. CD4+ cells were then cultured at a density of 10^6^ cells per well, in the presence of anti-CD3 monoclonal antibody (mAb) at 5µg/ml, anti-CD28 mAb at 3µg/ml, and IL-2 at 10ng/ml. Following four days in culture, the cells were supplemented with either 100 ng per well of CXCL9 or CXCL10 for 48 hours and subsequently analyzed via flow cytometry to assess the expression of CXCR3, PD-1, IFN-γ, Ki-67, IL-2, IL-17, granzyme B, and perforin. **(B)** CD4+ and CD8+ T cells activated with anti-CD3 from wild-type (WT) and CXCR3 knockout (KO) mice were assessed for the production of CXCL9 and CXCL10 by ELISA. **(C)** CXCL10 and CXCL9 drove the polarization of CD4+ T cells towards IFNγ^high^ Th1-like cells, Th17 effector cells, and granzyme-B high cytotoxic T cells, while also enhancing expression of Ki-67 and IL-2. Results present data of one of two independent experiments with similar observations. Significance was determined using a one-way analysis of variance (ANOVA) test using Tukey’s multiple comparisons test; *P≤ 0.05, **P≤ 0.01, ***P≤ 0.001, ****P<0.0001 were considered significant. ns, not significant.

**Figure 3:**
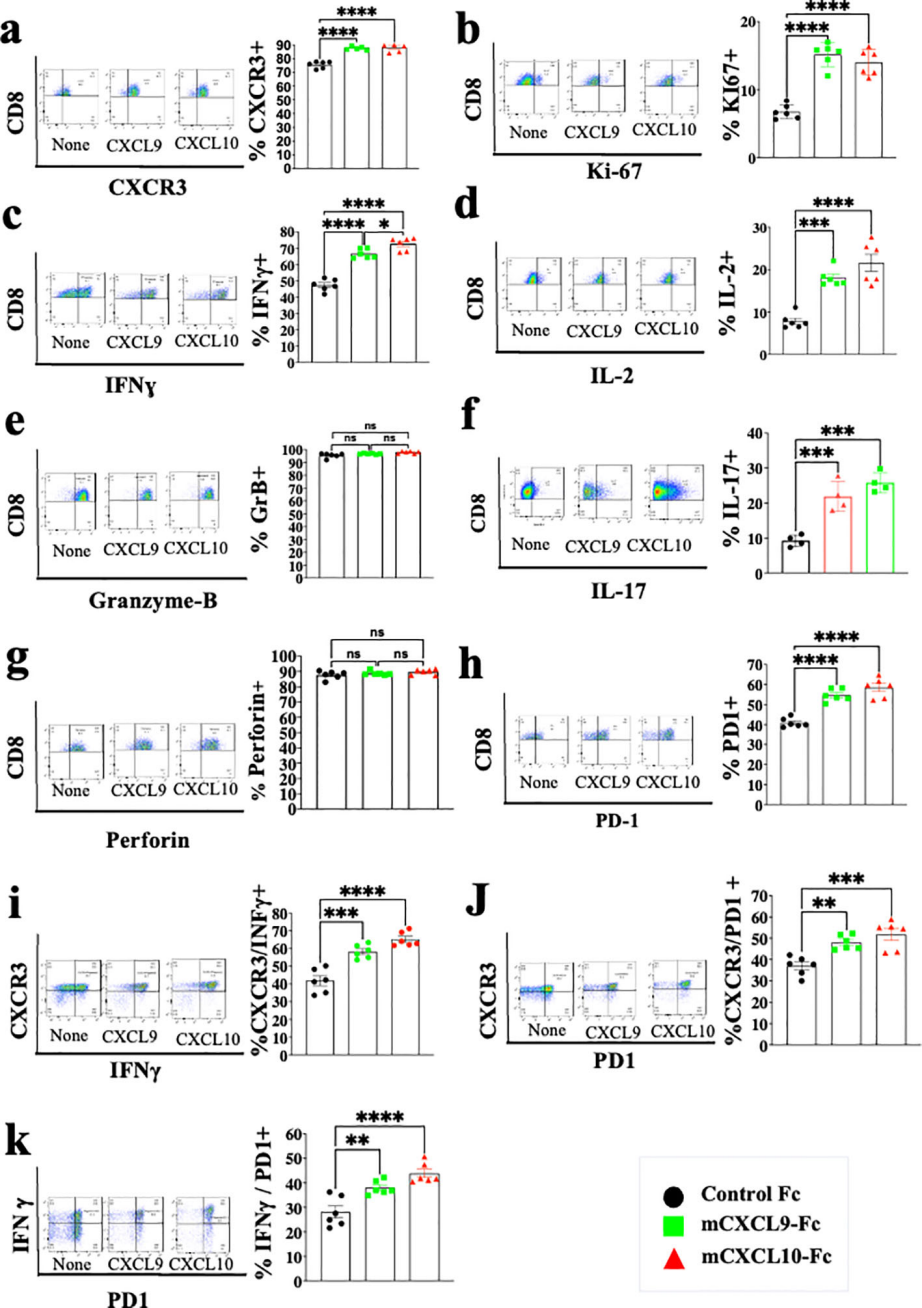
CXCL10 and CXCL9 drive the polarization of CD8+ T cells into IFNɣ^high^ cytotoxic T cells and Th17-like CD8+ T cells while increasing their Ki-67 and IL-2 expression. Spleenocytes were obtained from naïve CXCL10KO mice (6 per group) and sorted into CD8+ T cells using a MACS Easy-Sep magnetic separation kit. CD8+ T cells were then cultured at a density of 10^6^ cells per well, in the presence of anti-CD3 monoclonal antibody (mAb) at 5µg/ml, anti-CD28 mAb at 3µg/ml, and IL-2 at 10ng/ml. Following four days in culture, the cells were supplemented with either 100 ng of CXCL9 or CXCL10 for 48 hours and subsequently analyzed via flow cytometry to assess the expression of CXCR3, PD-1, IFN-γ, Ki-67, IL-2, IL-17, granzyme-B, and perforin. **(A)** Analysis of CXCR3 Vs. CD8 **(B)** Analysis of Ki67 Vs. CD8 **(C)** Analysis of IFN-γ Vs. CD8 **(D)** Analysis of IL-2 Vs. CD8 **(E)** Analysis of Granzyme-B Vs. CD8 **(F)** Analysis of IL-17 Vs. CD8 **(G)** Analysis of Peripherin Vs. CD8 **(H)** Analysis of PD-1 Vs. CD8 **(I)** Analysis of IFNγ Vs. CXCR3 **(J)** analysis of PD-1 Vs. CXCR3 **(K)** analysis of PD-1 Vs. IFNγ. Results present data from one of two independent experiments with similar observations Significance was determined using a one-way analysis of variance (ANOVA) test using Tukey’s multiple comparisons test; *P≤ 0.05, **P≤ 0.01, ***P≤ 0.001 were considered significant. ns, not significant.

Studies from various laboratories, including ours, previously suggested that CXCL10, and probably CXCL9, not only affect the migration of T cells, but also modulate their biological functions (22–27). A major limitation with all *in vitro* mechanistic studies thus far reported on the effects of CXCL9/10 on T cells relates to the fact that CXCL10 is produced to a varying degree by the cultured T cells undergoing activation, and CXCL9 may be produced by myeloid cells, when included. To avoid this complication, we generated an *in vitro* system in which the T cell response to exogenously added CXCR3 ligands could be measured in the absence of any endogenous CXCR3 ligand-production. We observed that under our working conditions, upon anti-CD3 & anti-CD28 mAb induced activation of purified T cells (see schematic diagram in **Figure 2A**), CD4+ and CD8+ T cells from WT C57BL/6 mice produced CXCL10, but not CXCL9, whereas CD4+ and CD8+ T cells from CXCL10KO mice did not produce CXCL10 and there was no compensation by CXCL9 (**Figure 2B**). C57BL/6 mice do not produce CXCL11 due to a natural mutation in the open reading frame of CXCL11 (36, 37). We therefore exploited this system, in which purified CD8+ T cells, or CD8+ T cells from CXCRL10LO mice were subjected to anti CD3&CD28 activation to analyze the differential contribution of CXCL9 and CXCL10 to the biological modulation of CXCR3+ T cells undergoing activation. **Figure 2C** summarizes the differential effect of CXCL10 and CXCL9 on cultured CD4+ T cells, whereas **Figure 3** shows the analysis of CD8+ T cells. The effect of either CXCL9 or CXCL10 on T cell polarization of CD4+ and CD8+ T cells was similar, with minor differences. For both, it included upregulation of Ki-67, IFN-γ and IL-2 (**Figures 2C**, **3B–D**), indicating a Th1-like CD4+ T cell and IFN-γ^high^ effector/cytotoxic CD8+ T cell polarization, activation and induction of proliferation. We also observed a significant increase in Th17-like CD4+ T cells, and IL-17-producing CD8+ T cells, also known as Tc17 cells (**Figures 2C**, **3F**), which are known to exhibit significant anti-tumor properties (38). Collectively, these data imply that both CXCL9 and CXCL10 induce the proliferation and increased effector function in both CD4+ T and CD8+ T cells, which is highly relevant for cancer therapy (39, 40). Notably, CXCL9 and CXCL10 induced IFN-γ in both CD4+ and CD8+ T cells. (panel c in **Figures 2**, **3**). Because CXCL10 is IFN-γ inducible (31, 32), it suggests a self-feeding loop that amplifies effector/cytotoxic T-cell functions. Further analysis showed that this interaction also upregulated the expression of PD-1 (panel h in **Figures 2**, **3**). The upregulation of PD-1 is likely to negatively control the positive feedback loop between CXCR3-CXCL9/CXCL10 and IFNγ; this may explain, in part, why anti-PD-1 ICT is more effective in CXCL9/CXCL10^high^ individuals. Moreover, a comparative analysis of IFN-γ upregulation and PD-1 expression showed their elevated expression in CXCR3+ T cells, as well as PD-1 expression on IFN-γ^high^ producing cells (**Figures 2**, **3I–K**).

### Crosstalk between cancer and the immune cells expands the self-feeding loop and its association with immune checkpoint blockade

**Figure 4:**
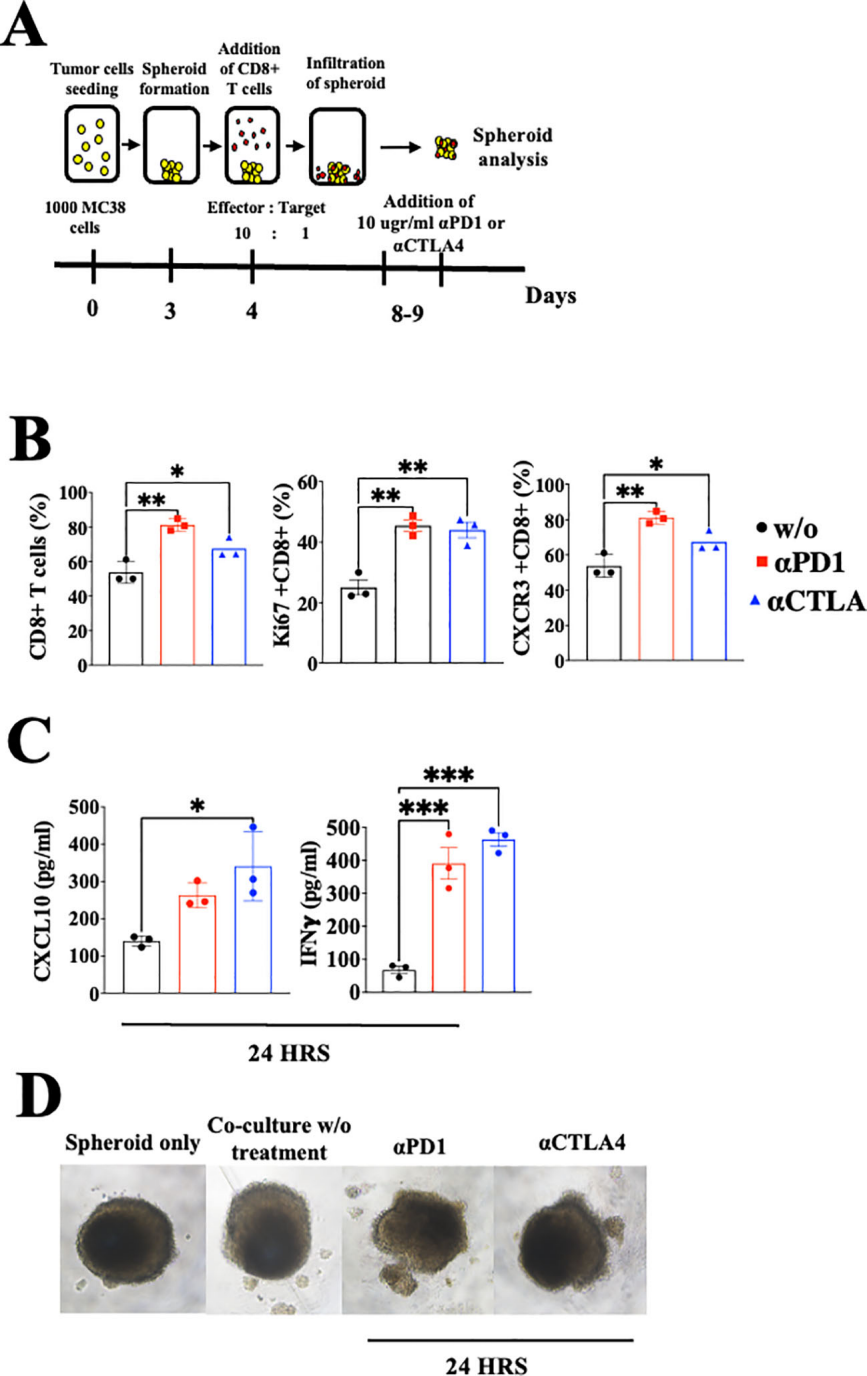
Blockade of PD-1 and CTLA-4 induces the CXCR3-CXCL10-IFNγ cycle in spheroids with activated CD8+ T cells coculture setup. **(A)** A scheme depicting the coculture protocol for MC38 spheroids with activated CD8+ T cells isolated from WT C57Bl/6 mice. MC38 spheroids were either cocultured with or without activated CD8+ T cells, supplemented with 10 µg/ml of αPD-1 or αCTLA4, and analyzed 24 hours post-treatment. **(B)** Flow cytometry analysis conducted on the interior compartment of the spheroid. **(C)** ELISA analysis of CXCL10 and IFN-γ levels following the addition of αPD-1 or αCTLA4. **(D)** Monitoring of spheroid structure using a light microscope. Results present data of one of three independent experiments with similar observations. Significance was determined using a one-way analysis of variance (ANOVA) test using Tukey’s multiple comparisons test; *P≤ 0.05, **P≤ 0.01, ***P≤ 0.001 were considered significant.

As previously addressed, CXCL10 is known to be IFN-γ-inducible (31, 32). To evaluate the possible extension of the CXCL10- IFN-γ axis to the cancer cells, we first evaluated whether IFN-γ also induced CXCL10 production in various murine and human cancer cell lines (**Supplementary Figure S1**). In four out of five different cell lines that we examined, IFN-γ significantly increased CXCL10 production but did not affect CXCL9 production. Notably, human A375 melanoma cells produced a similar base level of CXCL10 and CXCL9 (about 200 pg/ml) but in response to IFN-γ, it showed about a 6-fold increase in CXCL10 production, with no increase in CXCL9 production (**Supplementary Figure S1E**). To detect how anti-PD-1 or anti-CTLA-4 checkpoint blockade would interfere in the above cycle, we used a 3-D spheroid system (41) that includes tumor spheroids and CD8+ T cells from MC38 cancer-developing donors as described in **Figure 4A**. Cultures were or were not supplemented with 10 µg/ml αPD-1 or αCTLA4 and analyzed 24h later by flow cytometry and by ELISA (**Figures 4B, C**). The results clearly demonstrated that blockade of either PD-1 or CTLA-4 triggered the IFNγ-CXCL10-CXCR3 cycle that included a significant increase in the relative number of CD8+ T cells, their proliferation (Ki67+), CXCR3 expression, IFNγ production, and CXCL10 production. Collectively, this implies that ICT induces the IFN-γ-CXCL10-CXCR3 cycle, and its amplification is CXCL10-dependent. CXCL9 was not produced by CD8+ T cells or cancer cells and it has not been investigated as part of the loop, but should be taken into consideration at the tumor site where it is largely produced by CD106+ DC (19).

### Administration of CXCL10-Fc and CXCL9-Fc limits melanoma growth while selecting subtypes of effector and cytotoxic CD4+ and CD8+ T cells with an IFN-γ^high^ signature, further limiting tumor growth.

**Figure 5:**
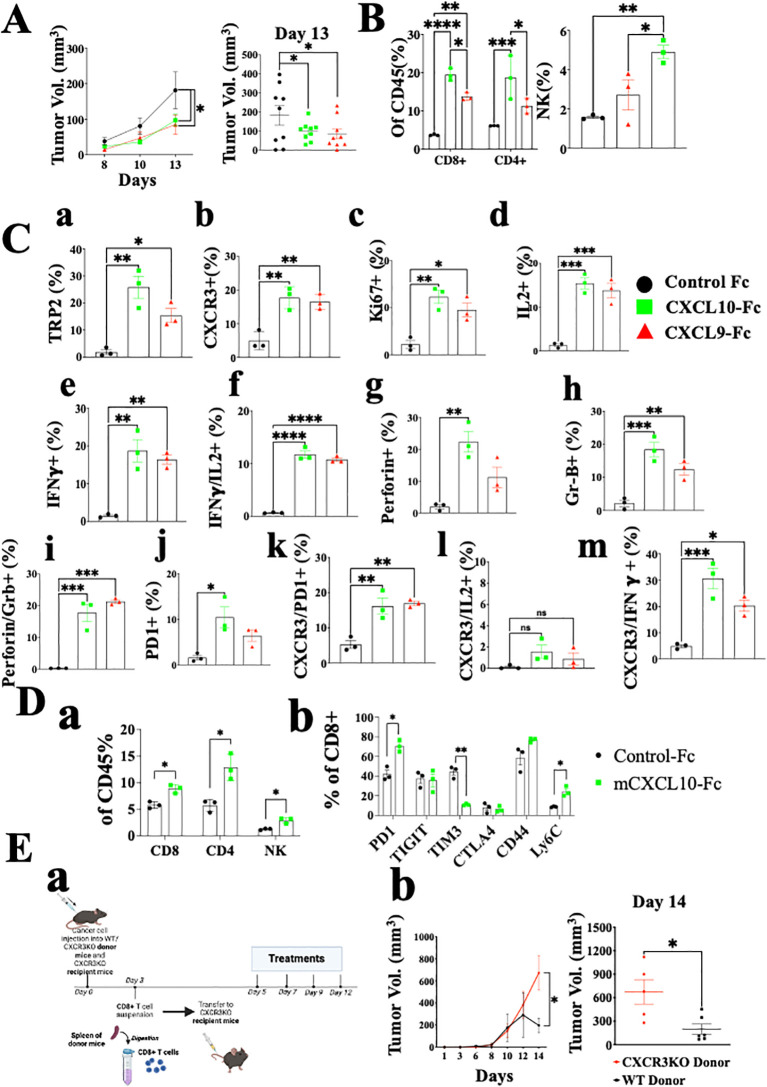
CXCL10-Fc and CXCL9-Fc induce IFNγ^high^, IL17^high^ effector/cytotoxic CD8+ T cells and exhaustion recovery that directly limit tumor growth C57BL/6 mice were subcutaneously injected with 2x10^5^ ret tumor cells overexpressing mCherry on the right flank. When tumor size reached about 50 mm^3^, they were separated into three identical groups of nine mice each. Each group was injected three times with 200µg/mouse CXCL10-Fc or CXCL9-Fc or with isotype matched control IgG, on days 8, 10, and 12. **(A)** The kinetics of tumor growth of all groups. **(B)** TILs were gated on CD45+ population. The effect of CXCL9-Fc or CXCL10-Fc therapy on the relative number of CD8+, CD4+, and NK cells at the tumor site is shown (as a percentage of total CD45+ population). **(C)** CD8+ T cell flow cytometry analysis (as a percentage of total CD45+CD8+ population) **(D** panel a) CXCL10-Fc directly induces CXCR3+ CD8+ T cells that restrain melanoma growth. **(D** panel b) PD-1, TIGIT, TIM3, CTLA4, CD44 and Ly6C expression determined on CD8+ T cells (as a percentage of total CD45+CD8+ population). **(E)** CXCL9-Fc limits tumor growth in CXCR3KO mice if reconstituted with CXCR3+ CD8+ T cells from cancer developing donors: (a) Schematic overview of the experimental protocol: Three days after subcutaneous engraftment of the ret melanoma cell line in either C57BL/6 WT or CXCR3 KO donor mice, CD8+ T cells were isolated from the spleen and intravenously transferred (0.5 × 10^6^ cells per mouse) into recipient mice lacking CXCR3. All mice received CXCL10-Fc treatment (40 µg/mouse) twice a week and were monitored for primary tumor development. (b) the kinetics of tumor development in each group and scattered analysis of tumor volume on the final day of the experiment, on day 14. Results represent data of one of three independent experiments with similar observations and are shown as mean ± standard deviation. Significance is determined using a one-way analysis of variance (ANOVA) test using Tukey’s multiple comparisons test; *P≤ 0.05, **P≤ 0.01, ***P≤ 0.001, ****P<0.0001 were considered significant.

Previously, we developed a fusion protein that includes CXCL10 linked to the N-terminus of murine IgG1 Fc (CXCL10-Fc) for cancer therapy and CXCL11-Fc for therapy of autoimmunity (27, 42). Each purified fusion protein was subjected to Western Blot analysis (**Supplementary Figure S2A**) and Ca++ flux using CHO cells overexpressing human CXCR3A (**Supplementary Figure S2B**). For *in vivo* studies, each fusion protein was administered to mice engrafted with the *ret* melanoma cell line. CXCL9-Fc and CXCL10-Fc significantly reduced tumor growth (**Figure 5A**, day 13 P<0.05). We analyzed the effect of therapy on the relative number (out of total CD45+ cells) of CD8+ T cells, CD4+ T cells, and NK cells at the tumor site. The relative number of CD8+ T cells increased from about 3% to 20% and 15% following CXCL9-Fc or CXCL10-Fc treatment, respectively; CD4+ T cells increased from about 5% to about 18% and 13%, respectively (**Figure 5B**). As for NK cells, their relative number in control mice was about 1.8% and increased about 2-fold in mice after CXCL10-Fc treatment (**Figure 5B**). Further analysis of CXCR3+ NK cells is beyond the scope of the current manuscript, which mainly focuses on CD8+ and CD4+ T cells.

As for CD8+ T cells, not only did their relative number dramatically increase, but also the relative number of tumor-specific T cells, as determined by TRP-2 pentamer-binding by tumor infiltrating lymphocytes (TILs), showed about 12-fold and a 7-fold increase in CXCL9-Fc and CXCL10-Fc treated mice, respectively (**Figure 5C**, a). A 3-fold increase was also observed in the expression of CXCR3 on these cells (**Figure 5C**, b). An about 5-fold increase in Ki67 accompanied by a similar increase in IL-2 production indicates an increase in the proliferation and activation state of these cells (**Figure 5C**, c, d). Most importantly, both CXCL9-Fc and CXCL10-Fc polarized T cells towards an IFN-γ high signature (**Figure 5C**, e). Further analysis of IFNγ *vs*. IL-2 (**Figure 5C**, f) indicates a clear association between increased IL-2 production and IFN-γ production in these cells. A very similar increase in perforin and granzyme-B was recorded in mice treated with CXCL9-Fc or CXCL10-Fc (**Figure 5C**, g, h). Further analysis of perforin ^high^ granzyme-B ^high^ CD8+ T cells (**Figure 5C**, i) show a clear association between increased perforin production and granzyme-B production in these cells. Thus, 20% of CD8+ T cells induced by CXCL9-Fc or CXCL10-Fc are cytotoxic CD8+ T cells, compared to less than 3% in control mice.

Finally, as a possible counter-mechanism, CXCL9-Fc and CXCL10-Fc led to a significant increase in PD-1 expression from about 2% to about 10% in CXCL9-Fc and about 7% in CXCL10-Fc treated mice (panel j). The significant increase in PD-1 expression, IL-2, and IFN-γ are all associated with increased CXCR3 expression on CD8+ T cells (**Figure 5C**, k–m). In conclusion, systemic administration of either CXCL9-Fc or CXCL10-Fc markedly increases the relative number of activated highly potent tumor-specific CD8+ T cells at the tumor site, and to some extent PD-1 expression on about 7-10% of these cells.

The possible association between CXCL9/CXCL10 interplay with CXCR3 and upregulation of PD-1 expression motivated us to explore further the effect of CXCL10-Fc on CTLA-4 and other inhibitory receptors including T cell immunoglobulin and mucin domain-containing protein 3 (TIM3) and tyrosine-based inhibition motif domain (TIGIT), which are also upregulated in exhausted T cells (43, 44). In addition, we examined the effect of CXCL10-Fc on the expression of CD44 and Ly6C, which are highly expressed on effector CD8^+^ T cells and, to a much lesser extent, on exhausted T cells (45). **Figure 5D** shows that CXCL10-Fc therapy led to a significant increase in the relative number of CD8+ T cells, CD4+ T cells, and NK cells at the tumor site (**Figure 5D**, a), and to a significant increase in PD-1 expression on these CD8+ T cells (**Figure 5D**, b); it also led to a marked decrease in TIM3 (p<0.001) and a significant increase in Ly6C (**Figure 5D**, b), with no significant effect in TIGIT. Collectively this implies that, despite the effect of CXCL10-Fc on the expression of PD-1, which may suggest shifting to T cell exhaustion (43, 44), all other markers imply that therapy may further induce effector/cytotoxic T cells. CTLA-4 expression was low in both groups and was not affected by therapy.

How significant is the direct effect of CXCL10-Fc on the ability of CXCR3+ CD8+ T cells to limit tumor growth? An adoptive transfer set-up in which CD8+ T cells were isolated from the spleen of either WT or CXCR3KO mice engrafted with *Ret* tumors, and transferred into *Ret*-tumor-harboring CXCR3KO recipient mice was conducted (**Figure 5E**, a). Both recipient groups were then treated with CXCL10-Fc. Only the administration of CXCL10-Fc to mice transferred with CXCR3+ CD8+ T cells led to a significant decrease in tumor size (**Figure 5E**, b). The results represent data from one of two independent experiments with very similar data. In conclusion, CXCL10, which directly polarizes and potentiates CXCR3+ CD8+ T cells (see **Figure 3**), also directly potentiates these cells to limit tumor growth.

**Figure 6:**
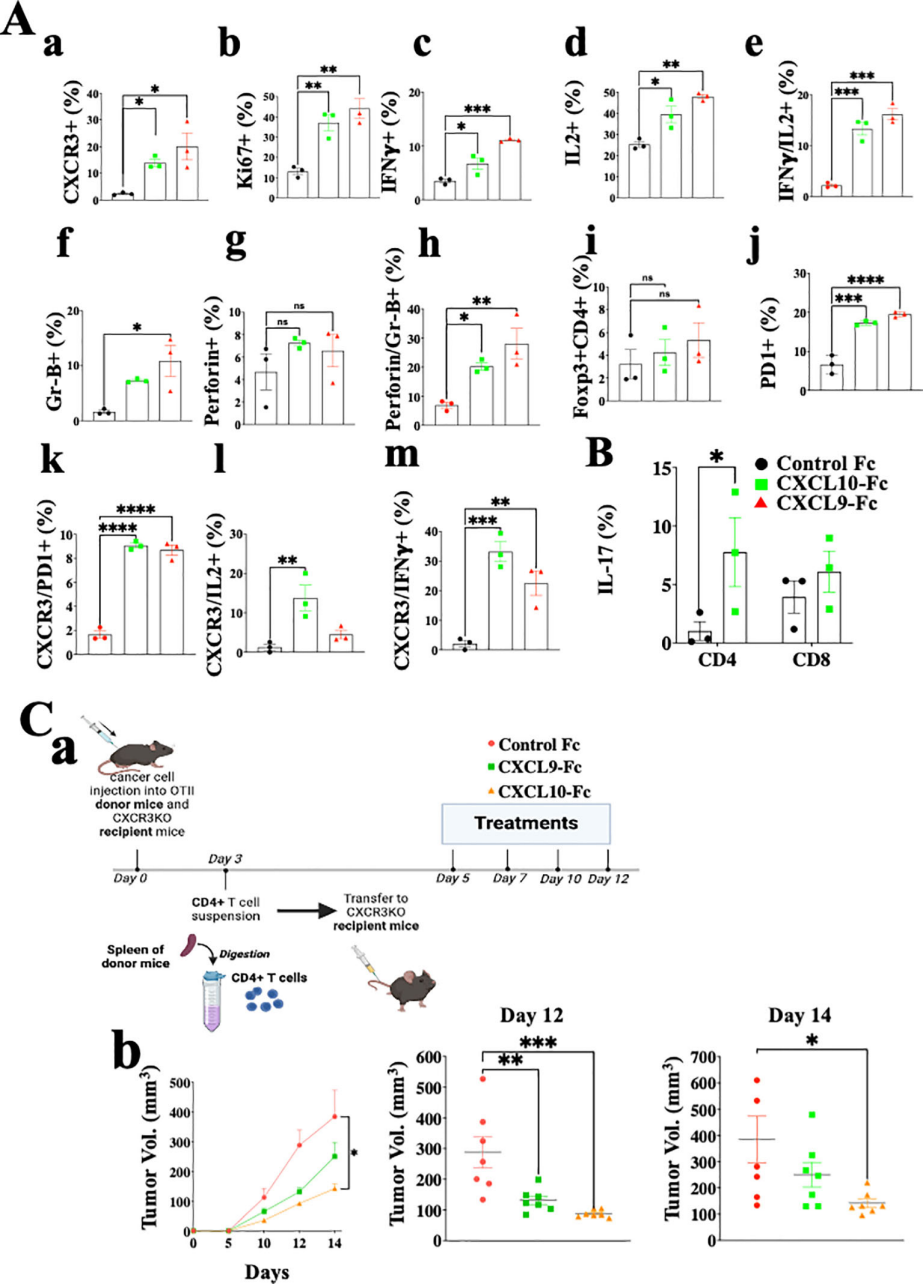
CXCL10-Fc and CXCL9-Fc induce IFNγ^high^ and IL17^high^ effector/cytotoxic CD4+ T cells that directly limit tumor growth. **(A)** CD4+ T cell analysis of the *ex vivo* experiment described in legend to **Figure 5**. **(B)** The effect of CXCL10-Fc therapy on the selection of IL-17^high^ CD45+CD4+ T cells **(C)** CXCL9-Fc and CXCL10-Fc limit tumor growth by directly affect CD45+CXCR3+ CD4+ T cells (a) Schematic overview of the experimental protocol: Three days after subcutaneous engraftment of the *ret* melanoma line overexpressing OVAII, either in OT-II donor mice or in CXCR3 KO recipient mice, CD4+ T cells were isolated from the spleen of OT-II donor mice and intravenously transferred (0.5 × 10^6^ cells per mouse) to three groups of CXCR3KO mice (7 females/group). Recipient mice were then administrated with CXCL9-Fc, CXCL10-Fc (twice a week, 50µg/mouse), or with isotype matched control IgG. The kinetics of tumor development in each group and scattered analysis of tumor volume on days 12 and 14. Results present data of one of three independent experiments with similar observations and are shown as mean ± standard deviation. Significance is determined using a one-way analysis of variance (ANOVA) test using Tukey’s multiple comparisons test; *P≤ 0.05, **P≤ 0.01, ***P≤ 0.001, ****P<0.0001 were considered significant. ns, not significant.

**Figure 6A** shows the analysis of CD4+ T cells in the same setup as described above for CD8+ T cells (**Figure 5C**). The results are very similar to those obtained for CD8+ T cells and include a highly significant increase in CXCR3 expression, about 6-fold following CXCL9-Fc therapy and about 10-fold following CXCL10-Fc therapy (**Figure 6**, a). A significant upregulation in T cell proliferation (Ki67), IFNγ, and IL-2 production was recorded (**Figure 6**, b–d). Further analysis of IFNγ *vs*. IL-2 (**Figure 6**, e) indicates a clear association between increased IL-2 production and IFN-γ production in these cells (Th1 polarization). Analyses of granzyme B (**Figure 6**, f) and perforin (**Figure 6**, g) shows a significant increase in granzyme B^high^ perforin ^high^ cytotoxic CD4+ T cells (**Figure 6**, h). The relative number of FOXp3+ Tregs was relatively low (about 3%) and gradually increased to about 4% following CXCL9-Fc and 5% following CXCL10-Fc therapy (**Figure 6**, i, nonsignificant). Similarly to CD8+ T cells, CD4+ T cells upregulated PD-1 the prevalence of expression from about 6% to 18% (**Figure 6**, j) with a high association with CXCR3 expression (**Figure 6**, k). We also analyzed the association between CXCR3 expression and either IL-2 or IFNγ expression. For both a highly significant increase in IL-2 and IFNγ was associated with the increase in CXCR3 expression (**Figure 6**, l, m). Collectively this further emphasizes the key role of CXCL9-Fc and CXCL10-Fc to induce IL-2^high^IFN^γhigh^ Th1cells.

In summary, the results of CD4+ T cell analyses are very similar to those obtained for CD8+ T cells and imply increased proliferation and shifting T cell polarization into IFN-γ high Th1-like cells and IFN-γ^high^ cytotoxic CD4+ T cells. In another experiment done using the same protocol, we evaluated IL-17 expression in CD4+ T cells and CD8+ T cells following CXCL10-Fc therapy (**Figure 6B**), revealing a significant increase in Th17-like CD4+ T cells.

To evaluate the significance of the direct effect of CXCL10-Fc on the ability of CXCR3+ CD4+ T cells to limit tumor growth, we performed an adoptive transfer in which CD4+ T cells were isolated from OT-II mice engrafted with *Ret* melanoma, transferred into CXCR3KO mice. This is an alternative protocol to the one used above for CD8 analysis (**Figure 5D**) and requires CXCR3KO OT-II donors. CD4+ T cells from OT-II mice (CXCR3+) engrafted with the *ret* melanoma cell line transduced to stably express OVA II (ret-OVAII melanoma cell line) were injected into CXCR3KO mice also engrafted with the ret-OVAII melanoma cell line and then treated with CXCL9-Fc, CXCL10-Fc or control IgG1 (Schematic View in **Figure 6C**, a) and monitored for tumor development. **Figure 6C**, b shows that both CXCL9-Fc and CXCL10-Fc significantly suppressed tumor development. CXCL10-Fc was superior to CXCL9-Fc (p<0.05). Collectively these results further highlight the role of CXCL9 and CXCL10 in potentiating, not only anti-tumor CD8+ T cells but also anti-tumor CD4+ T cells, including cytotoxic CD4+ T cells.

Collectively the above data imply that CXCL9 and CXCL10 directly polarize different subtypes of effector/cytotoxic T cells that are either CD4+ or CD8+, both with IFN-γ^high^ signature that can induce an IFN-γ dependent self-feeding loop to further amplify these activities. The results also indicate that the direct effect on either CD4+ or CD8+ T cells alone is sufficient to reduce tumor growth.

Finally, comparative analysis between the spleen and tumor cells of mice treated with either CXCL10-Fc or control IgG (**Supplementary Figure S3**) showed that successful therapy (**Supplementary Figure S3A**) led to a significant increase in CD4+ T cells and CD8+ T cells at the tumor site, but not spleen (**Supplementary Figure S3B**). Similarly, the relative number of NK cells also exclusively increased at the tumor site (**Supplementary Figure S3C**). Ki67 significantly increased in CD8+ (**Supplementary Figure S3D**) and CD4+ T cells (**Supplementary Figure S3F**) in both the spleen and the tumor site, indicating that CXCL10-Fc also induced these cells in the periphery, but the most significant result is at the tumor site where the relative tumor-specific CD8+ T cells are comparatively high (**Supplementary Figure S3E**). Intracellular cytokine staining further confirm the IFN-γ signature (**Supplementary Figure S3G**) described above. Finally, Ki67 staining of NK cells showed a significant increase at the tumor site, but not the spleen (**Supplementary Figure S3H**). It is, therefore, possible that the effect of CXCL10-Fc on NK cells is indirect and is in response to the high levels of IFN-γ, IL-2, and other cytokines at the tumor site.

### Exploring CXCL9-Fc and CXCL-10-Fc immunotherapy in immunocompetent Balb/C mice

**Figure 7:**
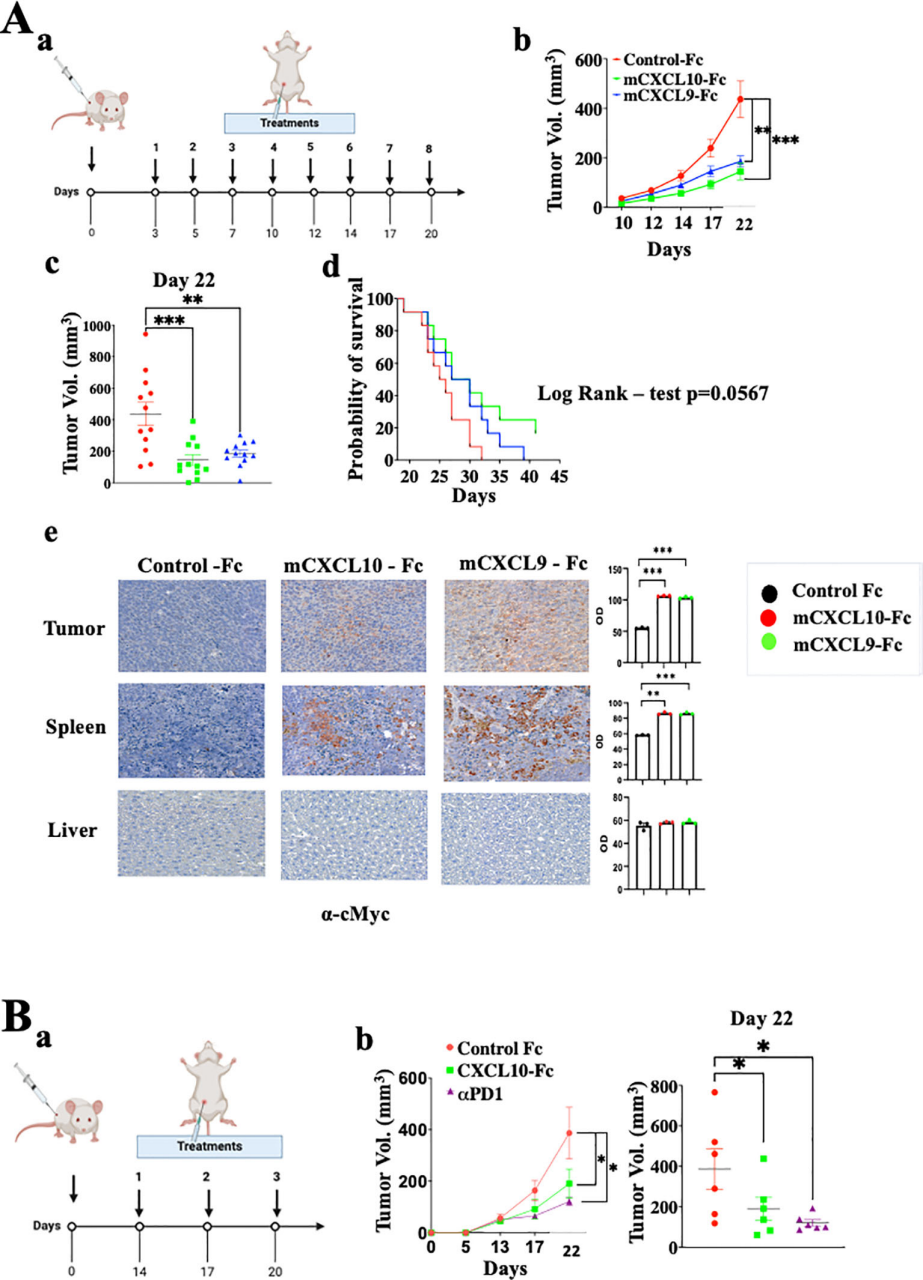
CXCL9-Fc and CXCL10-Fc limit tumor progression in CT26 colon cancer model. **(A)** Tumor progression kinetics in Balb/C mice treated with CXCL10-Fc or CXCL9-Fc. Balb/C mice were injected subcutaneously with 0.5x10^6^ CT26 cells at the right flank followed by treatment initiation with 50µg/mouse of either CXCL10-Fc or CXCL9-Fc three days post-tumor engraftment, along with a control group. (a) Dosing schedule: illustration of the treatment regimen. (b) Tumor progression kinetics. (c) Scattered analysis on day 22. (d) Survival curve with log rank test analysis. (e) Histology analysis of CXCL10-Fc, or CXCL9-Fc expression compared to control-Fc group using α-cMyc Ab. **(B)**Tumor progression rate in Balb/C Mice treated with CXCL10-Fc, or αPD-1. Balb/C mice were injected subcutaneously with 0.5x10^6^ CT26 cells at the right flank. When tumor size reached about 50 mm^3^ they were separated into three identical groups of six mice each and treated with 200µg/mouse of either CXCL10-Fc, 100µg/mouse of αPD-1, or with control Fc. (a) Dosing schedule: illustration of the treatment regimen. (b) Tumor progression kinetics. (c) Scattered analysis on day 22. Results of panel **(A, B)** are presented as mean ± standard deviation. Each represents two independent experiments with similar data. In each experiment scoring was done by an observer blind to the experimental protocol. Significance is determined using a one-way analysis of variance (ANOVA) test using Tukey’s multiple comparisons test; *P≤ 0.05, **P≤ 0.01, ***P≤ 0.001 were considered significant.

We further explored CXCL9-Fc and CXCL10-Fc therapy in the CT26 colon cancer model in WT Balb/C mice. These mice fully express functional CXCL9, CXCL10 and CXCL11. CXCL10 is also produced by the cancer cells (**Supplementary Figure S2C**) [see also (46)]. In the first set of experiments, a relatively low dose (50µg/mouse) of CXCL9-Fc or CXCL10-Fc was administered, starting 3 days after tumor engraftment (**Figure 7A**). Both agents significantly inhibited tumor growth (panels b and c, day 20 p<0.01) and increased survival (panel d) with an advantage to CXCL10-Fc (Log Rank – test p=0.0567**).** To follow the fate of CXCL9-Fc and CXCL10-Fc 24h post systemic administration, histological sections from the liver, spleen, or tumor site of representative mice injected with either control-Fc, CXCL10-Fc or CXCL9-Fc, were subjected to immunostaining with α-cMyc (included in the recombinant proteins), showing that CXCL9-Fc and CXCL10-Fc could be identified at the spleen and tumor site (where CXCR3+ T cells are present), but not in the liver (**Figure 7A**, e).

Finally, to further expand CXCL10-Fc therapy, it was administered three times, starting when tumor size reached vol of 50mm^3^. An anti-PD-1 mAb was used as a positive control. **Figure 7B** shows that both equally and significantly limited tumor growth.

## Discussion

The current study explores the interplay between CXCR3 and the two key immune checkpoint molecules, PD-1 and CTLA-4, aiming to elucidate the reports that checkpoint blockade is ineffective in the absence of CXCR3 (19, 47). Using an orthotopic model of melanoma, we observed that CXCR3KO mice displayed low responsiveness to anti-PD-1 and anti-CTLA-4 immunotherapy. Several recent studies further support these findings: Chow et al. showed, in a model of MC38 colon cancer, that CXCR3KO mice display low responsiveness to anti-PD-1 therapy (19); Ware et al. reported that the effect of anti- CTLA-4 combined with IL-6 therapy on pancreatic cancer is reduced when CXCR3 is blocked with an antibody (47). Finally, House et al. reported that antibodies to CXCR3 limit anti-CTLA-4 efficiency in the C57BL/6 mouse breast carcinoma following engraftment with AT-3 tumor line overexpressing OVA (48). Collectively these accumulating data indicate, not only a pivotal role of the CXCR3 axis in anti-cancer immunity, but also that one of the major mechanisms by which the immune ICB function is via the CXCR3 axis. This does not contradict the well-documented role of the PD-1 axis in regulating the transformation of exhausted T cells (T_EX_) into effector T cells (49–51). Anti-PD-1 therapy can convert T_EX_ into effector/cytotoxic T cells, thereby increasing the relative number of T cells that are potentiated via the CXCR3 cycle.

To explain the findings presented here and of others observing that CXCR3KO mice display reduced response to anti-PD-1 and anti-CTLA-4 checkpoint therapy (19, 47, 48), we suggest two different mechanisms that potentially occur simultaneously. The first, established in the current manuscript, suggests that one of the key roles of PD-1 and CTLA-4 is to regulate the CXCL10/CXC9-CXCR3 interplay that promotes effector/cytotoxic T cell functions in a CXCR3-CXCL10/CXCL9-IFNγ feedback loop. This may also explain why patients that are either CXCL9 or CXCL10 low are poor responders to anti-PD-1 therapy, as opposed to those that are CXCL9 or CXCL10^high^ (13–19). A second possible mechanism relates to crosstalk between the PD-1 or CTLA-4 axis and the CXCR3 axis. The latter needs to be further explored.

The two complementary key questions that the current manuscript asks are: (1) What is the mechanism by which CXCL9 and CXCL10 potentiate effector/cytotoxic CD4+ and CD8+ T cells? and (2) whether the administration of CXCL9-Fc or CXCL10-Fc could amplify these effects The second question holds much relevance for combination therapy consisting of ICT and CXCL9-Fc/CXCL10-Fc. The observations that PD-1 and CTLA-4 are key regulators of the CXCR3-CXCL9/CXCL10 axis, which may explain in part why patients that are either CXCL9 or CXCL10 low are poor responders to anti-PD-1 therapy, as opposed to those that are CXCL9 or CXCL10^high^, could have clinical implications for personalized therapy, and may extend the degree of responses to these immune checkpoint inhibitors. The relevance of the CXCR3-CXCL9/CXCL10 axis to other ICT including TIGIT, LAG-3, and TIM-3 has yet to be studied.

The most relevant human cancers in this context are melanoma, non-small cell lung cancer (NSCLC), ovarian cancer, gastric cancer, and colorectal cancer, where high levels of CXCL9/CXCL10 indicate favorable prognosis and low levels poor prognosis (13–19). The response to ICT in melanoma patients correlates to CXCL9/10 expression levels in blood or at the tumor site) (18, 19). Likewise, NSCLC patients with high plasma levels of CXCL9 and CXCL10 displayed better responses to anti-PD-1 or anti-PD-L1 therapy (13). It should be noted that some melanoma patients receive combination treatments with anti-CTLA-4 and anti-PD-1 mAbs, and yet almost 50% of them show negligible responses (2). It is possible that adding CXCL9-Fc/CXCL10-Fc to the existing antibody therapy would enhance responses in these patients. One possibility for identifying candidates for CXCL0-Fc or CXCL10-Fc therapy would be to culture patient biopsies (52, 53) and examine whether the addition anti-PD-1 or anti-CTLA-4 mAbs increase CXCL10 and CXCL9 levels, using an experimental set-up similar to that represented in **Figure 4**.

Our data suggest a potential use of CXCL9-Fc and/or CXCL10-Fc for cancer immunotherapy. CXCL9-Fc and CXCL10-Fc could be administered i.v., for example, during early clinical development stages. In our study, repeated administration was i.p. Future studies will compare the two routes of administration. One of the potential limitations of biological drugs that enhance anti-tumor immunity is the risk of toxicity. This issue can be carefully addressed in pre-clinical studies. One optional possibility that would be considered in case of toxicity is mutating CXCL10 and the development of less toxic variants as has recently been done for IL-2 (54).

The *in vitro* studies presented in **Figures 2**, **3** and the *ex vivo* analyses presented in **Figures 5**, **6** show that the direct effects of CXCL9 and CXCL10 include skewing of CD4+ and CD8+ T cell polarization into IFN-γ^high^ T cells, including IL17^high^ CD4+ T cells (Th17) and IL17^high^ CD8+ T cells (significant in the in *ex vivo* model), also known as Tc17 cells, which are thought to play a major role in combating cancer (38). Both CXCL9 and CXCL10 induced rapid proliferation (ki-67) and IL-2 production by both CD4+ and CD8+ T cells, with a marked increase in tumor-specific (TRP2-specific) CD8+ T cells, and an increase in granzyme-B+ CD4+ T cells, which may explain in part the efficacy of the adoptive transfer studies in which CXCL10-Fc and CXCL9-Fc limit tumor progression in CXCR3KO mice that are reconstituted with CXCR3+ CD4+ T cells (**Figure 6B**). Such cytotoxic CD4+ T cells appear to play a key role in limiting tumor progression, particularly those in which tumor antigens are presented by MHCII on cancer cells, among them bladder cancer (55) and melanoma (56). Likewise, the adoptive transfer of CXCR3+ CD8+ T cells into CXCR3KO mice enabled response to CXCL10-Fc (**Figure 5E**), showing that the direct effect on CD8+ T cells induced by CXCL10-Fc is sufficient to restrain tumor growth.

The increased expression of PD-1 on both CD4+ and CD8+ T cells in cultures supplemented with CXCL9 or CXCL10, and upregulation of PD-1 on these cells *in vivo* following CXCL9-Fc and CXCL10-Fc therapy, may suggest that the CXCL9/CXCL10 interaction with CXCR3 that potentiate effector/cytotoxic T cells is partially regulated by this increase. The increased expression of PD-1 following CXCL10-Fc therapy is not associated with any increase in other inhibitory receptors including HAVCR2 (TIM3) and TIGIT, that may indicate increase in CD8+ T cell exhaustion (43, 44), indicated that. In addition, we examined the effect of CXCL10-Fc on the expression of CD44 and Ly6C, which are highly expressed on effector CD8^+^ T cells, and to a much lesser extent on exhausted T cells (45). Collectively our results show that the chemokine therapy described here does not increase T cell exhaustion, but on the contrary, may further induce effector/cytotoxic T cells. Notably, these therapies led to a marked decrease in TIM3, which may be part of the mechanism of T cell potentiation.

According to our results it is likely that CXCL9 and CXCL10 possess very similar activities, differing mainly by the cells that produce them. CXCL9 is mainly produced by macrophages and DC, particularly CD106+ DC (19), whereas CXCL10 is mainly expressed by effector T cells and, in a wide range of tumors, by the cancer cells themselves. The importance of CXCL10 produced by the cancer cells has been emphasized by a recent study comparing response to anti-PD-1 using colon cancer cells that are CXCL10^high^ (CT26), and CXCL10low (Colon 26), showing that only the first respond to anti-PD-1 treatment (46). Furthermore, CXCL10KO mice did respond to anti-PD-1 when the engrafted tumor cells were CXCL10high (MC38) (19).

Collectively, this manuscript suggests that the CXCL9/10-CXCR3 axis is important, not merely in the trafficking of T cells to tumors (57), but even more so in the polarization, potentiation, and proliferation of these cells, and the self-feeding loop that further promotes their activities. This may also explain why peripheral (systemic) administration of CXCL9-Fc or CXCL10-Fc limits tumor growth even though systemic administration of these agents would be expected to compromise the chemokine gradients that allow the endogenous CXCR3 ligands to attract T cells into the tumors. Finally, and most importantly, the manuscript brings compelling evidence implying a pivotal role for the PD-1 and CTLA-4 axes in regulating the CXCR3/CXCL9/10, leading to the induction of effector/cytotoxic T cells. The role of other known ICI in the regulation of this axis has yet to be studied.

## Materials and methods

### Mouse strains and maintenance

In all experiments, 8-10 weeks old female mice were used. C57BL/6 (WT) and Balb/C (WT) mice were purchased from Harlan (Israel). CXCR3KO mice, CXCL10KO mice, OTII mice, and OTI mice were purchased from JAX lab (Bar Harbor, Maine). All mice were maintained under individually ventilated caging systems (IVC). All experiments were approved by the Technion Committee for Care and Use of Laboratory Animals which operates under the NIH guideline (Technion animal experimentation protocol No: IL-072-05-2021, valid until June 2025).

### Cell lines and culture

Ret (murine melanoma cell line) was kindly provided by Prof. Neta Erez from Tel Aviv University, with permission of Prof. Viktor Umansky (DKFZ). CT26 (murine colon cancer cell line) and MC38 colon cancer cell line were purchased from ATCC. Cells were routinely tested for mycoplasma contamination and maintained under 37°C and 5% CO_2_ conditions in RPMI Medium 1640 (Gibco, Rhenium, Israel, Cat. 21875034). Cho-Ki cells were purchased from Perkin Elmer (USA) and maintained under 37°C and 8% CO_2_ conditions in F-12 (Nutrient Mixture F-12(Ham), Gibco, Rhenium, Israel, Cat. 21765029). All media were supplemented with 10% fetal calf serum (Fetal Bovine Serum Heat Inactivated, Merck Life Science, Israel, Cat. F9665-500ML), 1% L-glutamine (Gibco, Rhenium, Israel, Cat. 25030024), and 1% Pen-Strep solution (Gibco, Rhenium, Israel, Cat. 15140122).

### Tumor engraftment

Ret and CT26 cells were collected after trypsinization, washed with PBS, and resuspended in 100µl PBS per mouse (4.5x10^5^/100μL in PBS). Cells were injected subcutaneously into the right flanks of 6–8 week-old female C57BL/6, or BALB/c mice. In all experiments, when tumors reached a volume of ∼50 mm^3^, mice were randomized into different experiment groups, monitored daily for evidence of illness, and treated with mouse anti-PD-1 (clone RMP1-14, BioXCell Cat. BE0146), mouse anti-CTLA-4 (clone 9H10, BioXCell Cat. BE0131) antibodies or mouse CXCL10-Fc and CXCL9-Fc proteins. Control groups were treated with IgG isotype control (BioXCell Cat. BE0089). Tumor volume was measured manually using an electronic caliper. Tumor volume was calculated using the formula π/6 × a × b × c, where a represents the length, b represents the width, and **c** represents the height of the tumor (58).

### Western blotting

Protein samples were separated on 4%-20% SDS-PAGE mini gels (GeneScript) and transferred to nitrocellulose membranes using a semi-dry blotter (Trans blot Turbo transfer system, Biorad) at 1.3A/25V per mini gel for 7 min, in transfer buffer (250mM Glycine, 25mM Trizma base, 20% methanol). The membrane was blocked with 1% BSA in PBS for 1h at room temperature (RT) and incubated with 1:5000 anti-HIS tag HRP-conjugated primary antibody, diluted in blocking solution. The membrane was shaken for 2 hours at RT, followed by three washes with TBST (150mM NaCl, 20mM Tris-HCl pH=8, 0.1% Tween 20), incubated for 1 min in EZ-ECL solution (Advansta K-12045-D20) and developed on VILBER Fusion FX7 Machine.

### Cytokines quantification by indirect ELISA (sandwich)

Cytokine concentrations in culture media were assessed using commercial ELISA kits: Murine IP-10 (CXCL10) Standard ABTS ELISA Development Kit, (900-K153, Peprotech), Human IP-10 (CXCL10) Standard ABTS ELISA Development Kit (900-K39, Peprotech), ELISA Kit for MOUSE CXCL9 (UCSN SEB928Mu), Human MIG (CXCL9) Standard ABTS ELISA Development Kit (900-K87, Peprotech) and Murine IFN gamma Standard ABTS ELISA Development Kit, (900-K98, Peprotech) according to manufacturer’s instructions.

### Spleenocyte isolation and activation

CD4^+^ and CD8^+^ T cells were isolated from whole spleens by EasySep™ CD4^+^ (StemCell Technologies, Cat. 19852A) or CD8^+^ (StemCell Technologies, Cat. 19853A) T cells enrichment magnetic beads according to manufacturer standard protocol. Spleenocytes were activated with 5 µgr/ml of anti-mouse CD3ϵ (Biolegend Cat. 100302), 3 µg/ml of anti-mouse CD28 (Biolegend Cat. 102116) and 10 ng/ml of Murine IL-2 (Peprotech Cat. 212-12-20). They were separately cultured *in vitro* in DMEM media.

### TIL isolation

TIL isolation was performed as follows: 1 gram of tumor tissue was cut into ≤ 5mm pieces, transferred into a GentleMacs tube (Cat. 130-096-334 purple cap) containing 5ml of cold RPMI 1640 medium, supplemented with 20% FCS, 2% P/S and run on the GentleMacs device, using the program: m_impTumor_02*, for three times. 75 µl of freshly prepared collagenase I solution (Sigma, Cat. c0130) and 150 µl Dispase II solution (Roche, Cat. 04942078001) were added to the minced tissue and incubated at 37°C, on a shaker 110rpm, 40min. The tubes were run again on the GentleMacs device, using the program: m_impTumor_04*, three times.

The liquid was passed through a 40µm cell strainer (Bar Naor, Cat. BN93040S), washed with cold PBSx1, and completed the volume to 25ml with cold PBSx1 in a 50 ml tube. The solution was centrifuged at 1500 rpm for 5 min at 4°C and then resuspended with 5 ml of cold RBC lysis (41.4 gr NH_4_Cl buffer, 5 gr NaHC0_3_, 0.189 gr EDTA, 500 ml DDW), vortexed and incubated for 7 min at RT. 45 ml of cold PBSX1 was added to the tube, centrifuged at 1500 rpm, for 5 min, at 4°C, and then resuspended with the FACS buffer (500ml PBSX1, 10 ml FCS, 10 ml 50Mm EDTA)/medium for further procedure. Spleenocyte isolation was performed as follows: a spleen was minced in 10 ml of cold PBSX1 using a 40µm cell strainer (Bar Naor, Cat. BN93040S), centrifuged at 1500 rpm, for 5 min, at 4°C and resuspended with 0.5 ml of cold RBC lysis buffer (Rhenium, Cat. 00-4333-57), for 20 seconds at RT. The final volume was completed to 10 ml with PBSX1 and centrifuged again under the same conditions. The pellet was resuspended in 2 ml of FACS buffer for further analysis.

### Flow cytometry acquisition and analysis

Before immunostaining, cells were plated at a density of 1x10^6^ cells/well in an appropriate medium and stimulated for 4-6 hours under 37°C and 5% CO_2_ conditions with Cell Activation Cocktail (Biolegend 423303). Tumor and spleen cells were immuno-stained for the following surface and intracellular markers. Cell permeabilization was carried out using the BD Biosciences kit (BD Biosciences 554714) according to the manufacturer’s instructions. All monoclonal antibodies were purchased from BD Biosciences and BioLegend. Flow cytometry data was acquired on BD LSRFortessa using and analyzed with FlowJo V.10 software (FlowJo, Ashland, Oregon, USA).

### Paraffin embedding of tumor, spleen, and liver tissues

Tumor and spleen tissues were immediately fixated in 4% formaldehyde PH=7.2 overnight, the next day they were placed in tissue cassettes and kept in ethanol 70% overnight. The next day, tumor tissue was subjected to the processes of dehydration - three exchanges of ethanol 95% (20min each), three exchanges of ethanol 100% (20min each), clearing - two exchanges of chloroform (10min each), and embedding - two exchanges of paraffin (1h each, 60°C), followed by a third exchange of paraffin overnight. Tumor tissue was then molded into paraffin blocks and 5µm sections were cut, and were let dry overnight at 37°C until stained.

### Immunohistological staining

Slides were de-paraffinized at 60°C for 1 hour and incubated in K-Clear solution for 2 repeats of 5 min each and in 100% EtOH for 2 repeats of 5 min each. Endogenous peroxidase blocking was performed using freshly prepared 100% methanol with 1% H_2_O_2_, followed by 1-2 min wash in 70% EtOH and 3x rinsing with double distilled water. (DDW). Antigen retrieval was performed by microwave boiling the slides in 10 sodium citrate buffer, pH 6 for 22 minutes. The slides were slowly cooled down to RT and washed twice with PBS. Blocking was performed using 10% goat serum for 1 hour at RT. Following blocking, slides were incubated with primary antibodies overnight, at 4°C. On the next day, the slides were washed 4x5 min in PBS. 2-3 drops of HISTOFINE Simple Stain Max Po (Multi) Universal Immuno peroxidase Polymer anti–rabbit/mouse (Nichirei) were added followed by 1-hour incubation, RT. Slides were then washed 4x5 min in PBS and then incubated with HISTOFINE Simple Stain AEC Solution (10 min, RT) (Nichirei), and then washed until clear and then counter-stained for 30 sec with hematoxylin. Slides were then washed and left in DDW for 10 min at RT, and then mounted and sealed with mounting medium (Immuno Mount, Thermo Shandon). Statistical analysis was performed using Fiji software.

### Calcium assay

Calcium assay was performed in CHO cells which overexpress human CXCR3A. GPCR stimulation with a CXCR3A ligand induces Ca++ flux. The assay is based on a reporter system that includes calcium binding to the aequorin oxidation of coelenterazine which leads to the emission of light (469nm). Ca++ flux in CHO cells was induced by 100 ng of CXCL10 (Peprotech) and CXCL9 (Peprotech) chemokines and detected using the Calcium Assay Kit (Zotal, AB-ab112114-10). The OD measurements were performed on a plate reader (Infinite M200 PRO).

### Adoptive transfer protocol

Three days after the *ret* melanoma line was engrafted into either WT or CXCR3KO donor mice, CD8+ T cells were isolated from the spleen and transferred (0.5X10^6^ cells per mouse) into CXCR3KO mice 3 days following engraftment with the *ret* melanoma line. All mice were treated twice a week with mCXCL10-Ig (200µg/mouse) and monitored for primary tumor development. On day 20, mice were sacrificed, and tumor weight was measured.

### Expression and purification of fusion proteins

Fusion proteins were expressed using the Expi293™ Expression System according to the manufacturer’s protocol (Thermo Fisher Scientific, Cat. Number A14635, Publication Number MAN0019402) and purified on an Ni-NTA agarose column (QIAGEN, Cat. Number 30210).

### Spheroids

MC38 spheroids were generated by seeding 1,000 cells per well on Nunclon Sphera (ThermoFisher) round bottom 96 wells plates in complete DMED medium. Four days later, cocultures were started by adding 10x10^3^ total of activated CD8+ T cells extracted from WT C57Bl/6 mice, per well; 24 hours later, 10 µg/ml of anti-PD-1 or anti CTLA4 or PBS were added to each well. For flow cytometry analyses, 12 wells per condition were seeded and divided into 4 groups. Spheroids were isolated from wells, gently resuspended, and trypsinized to obtain a single-cell suspension before further analysis by flow cytometry.

### Statistical analysis

Statistical analyses were performed using GraphPad Prism software version 8.0. For comparison, two samples mean t-test was used, and for multiple experiments, the statistical method of choice was the one-way analysis of variance (ANOVA) test using the Tukey multiple comparisons test. Log-rank test was used to compare the survival distributions of two samples. *P*< 0.05 was considered statistically significant.

## Data Availability

The original contributions presented in the study are included in the article/**Supplementary Material**. Further inquiries can be directed to the corresponding author.

## References

[1] Sharma P Goswami S Raychaudhuri D Siddiqui BA Singh P Nagarajan A. Immune checkpoint therapy-current perspectives and future directions. Cell. (2023) 186:1652–69. doi: 10.1016/j.cell.2023.03.006 37059068

[2] Larkin J Chiarion-Sileni V Gonzalez R Grob JJ Cowey CL Lao CD. Combined nivolumab and ipilimumab or monotherapy in untreated melanoma. N Engl J Med. (2015) 373:23–34. doi: 10.1056/NEJMoa1504030 26027431 PMC5698905

[3] Colvin RA Campanella GS Sun J Luster AD. Intracellular domains of CXCR3 that mediate CXCL9, CXCL10, and CXCL11 function. J Biol Chem. (2004) 279:30219–27. doi: 10.1074/jbc.M403595200 15150261

[4] Xiu W Luo J. CXCL9 secreted by tumor-associated dendritic cells up-regulates PD-L1 expression in bladder cancer cells by activating the CXCR3 signaling. BMC Immunol. (2021) 22:3. doi: 10.1186/s12865-020-00396-3 33407095 PMC7789583

[5] Oghumu S Varikuti S Terrazas C Kotov D Nasser MW Powell CA. CXCR3 deficiency enhances tumor progression by promoting macrophage M2 polarization in a murine breast cancer model. Immunology. (2014) 143:109–19. doi: 10.1111/imm.2014.143.issue-1 PMC4137960 24679047

[6] Singh UP Singh R Singh S Karls RK Quinn FD Taub DD. CXCL10+ T cells and NK cells assist in the recruitment and activation of CXCR3+ and CXCL11+ leukocytes during Mycobacteria-enhanced colitis. BMC Immunol. (2008) 9:25. doi: 10.1186/1471-2172-9-25 18533024 PMC2443107

[7] Hansen DS Bernard NJ Nie CQ Schofield L. NK cells stimulate recruitment of CXCR3+ T cells to the brain during Plasmodium berghei-mediated cerebral malaria. J Immunol. (2007) 178:5779–88. doi: 10.4049/jimmunol.178.9.5779 17442962

[8] Xu J Fu H Yang Y Yu H Ai X Lei Y. Modulation of CXCR1 and CXCR3 expression on NK cells via Tim-3 in a murine model of primary biliary cholangitis. Mol Immunol. (2021) 135:342–50. doi: 10.1016/j.molimm.2021.04.014 33984607

[9] Fenwick PS Macedo P Kilty IC Barnes PJ Donnelly LE. Effect of JAK inhibitors on release of CXCL9, CXCL10 and CXCL11 from human airway epithelial cells. PloS One. (2015) 10:e0128757. doi: 10.1371/journal.pone.0128757 26090665 PMC4474874

[10] Li CX Ling CC Shao Y Xu A Li XC Ng KT. CXCL10/CXCR3 signaling mobilized-regulatory T cells promote liver tumor recurrence after transplantation. J Hepatol. (2016) 65:944–52. doi: 10.1016/j.jhep.2016.05.032 27245433

[11] Paust HJ Riedel JH Krebs CF Turner JE Brix SR Krohn S. CXCR3+ Regulatory T cells control TH1 responses in crescentic GN. J Am Soc Nephrol. (2016) 27:1933–42. doi: 10.1681/ASN.2015020203 PMC4926966 26534920

[12] Redjimi N Raffin C Raimbaud I Pignon P Matsuzaki J Odunsi K. CXCR3+ T regulatory cells selectively accumulate in human ovarian carcinomas to limit type I immunity. Cancer Res. (2012) 72:4351–60. doi: 10.1158/0008-5472.CAN-12-0579 22798340

[13] Eltahir M Isaksson J Mattsson JSM Karre K Botling J Lord M. Plasma proteomic analysis in non-small cell lung cancer patients treated with PD-1/PD-L1 blockade. Cancers (Basel). (2021) 13:1–17. doi: 10.3390/cancers13133116 PMC8268315 34206510

[14] Bronger H Singer J Windmuller C Reuning U Zech D Delbridge C. CXCL9 and CXCL10 predict survival and are regulated by cyclooxygenase inhibition in advanced serous ovarian cancer. Br J Cancer. (2016) 115:553–63. doi: 10.1038/bjc.2016.172 PMC4997538 27490802

[15] Chen J Chen QL Wang WH Chen XL Hu XQ Liang ZQ. Prognostic and predictive values of CXCL10 in colorectal cancer. Clin Transl Oncol. (2020) 22:1548–64. doi: 10.1007/s12094-020-02299-6 32016676

[16] Ardighieri L Missale F Bugatti M Gatta LB Pezzali I Monti M. Infiltration by CXCL10 secreting macrophages is associated with antitumor immunity and response to therapy in ovarian cancer subtypes. Front Immunol. (2021) 12:690201. doi: 10.3389/fimmu.2021.690201 34220848 PMC8253056

[17] Huang J Song J Li X Liu S Huang W Shen Z. Analysis and prognostic significance of tumour immune infiltrates and immune microenvironment of m6A-related lncRNAs in patients with gastric cancer. BMC Med Genomics. (2022) 15:164. doi: 10.1186/s12920-022-01318-5 35879790 PMC9310490

[18] Reschke R Yu J Flood B Higgs EF Hatogai K Gajewski TF. Immune cell and tumor cell-derived CXCL10 is indicative of immunotherapy response in metastatic melanoma. J Immunother Cancer. (2021) 9:1–8. doi: 10.1136/jitc-2021-003521 PMC8487215 34593622

[19] Chow MT Ozga AJ Servis RL Frederick DT Lo JA Fisher DE. Intratumoral activity of the CXCR3 chemokine system is required for the efficacy of anti-PD-1 therapy. Immunity. (2019) 50:1498–1512.e5. doi: 10.1016/j.immuni.2019.04.010 31097342 PMC6527362

[20] Seitz S Dreyer TF Stange C Steiger K Brauer R Scheutz L. CXCL9 inhibits tumour growth and drives anti-PD-L1 therapy in ovarian cancer. Br J Cancer. (2022) 126:1470–80. doi: 10.1038/s41416-022-01763-0 PMC9090786 35314795

[21] Liang YK Deng ZK Chen MT Qiu SQ Xiao YS Qi YZ. CXCL9 is a potential biomarker of immune infiltration associated with favorable prognosis in ER-negative breast cancer. Front Oncol. (2021) 11:710286. doi: 10.3389/fonc.2021.710286 34527583 PMC8435794

[22] Dufour JH Dziejman M Liu MT Leung JH Lane TE Luster AD. IFN-gamma-inducible protein 10 (IP-10; CXCL10)-deficient mice reveal a role for IP-10 in effector T cell generation and trafficking. J Immunol. (2002) 168:3195–204. doi: 10.4049/jimmunol.168.7.3195 11907072

[23] Gangur V Simons FE Hayglass KT. Human IP-10 selectively promotes dominance of polyclonally activated and environmental antigen-driven IFN-gamma over IL-4 responses. FASEB J. (1998) 12:705–13. doi: 10.1096/fasebj.12.9.705 9619449

[24] Wildbaum G Netzer N Karin N. Plasmid DNA encoding IFN-gamma-inducible protein 10 redirects antigen-specific T cell polarization and suppresses experimental autoimmune encephalomyelitis. J Immunol. (2002) 168:5885–92. doi: 10.4049/jimmunol.168.11.5885 12023393

[25] Salomon I Netzer N Wildbaum G Schif-Zuck S Maor G Karin N. Targeting the function of IFN-gamma-inducible protein 10 suppresses ongoing adjuvant arthritis. J Immunol. (2002) 169:2685–93. doi: 10.4049/jimmunol.169.5.2685 12193742

[26] Groom JR Richmond J Murooka TT Sorensen EW Sung JH Bankert K. CXCR3 chemokine receptor-ligand interactions in the lymph node optimize CD4+ T helper 1 cell differentiation. Immunity. (2012) 37:1091–103. doi: 10.1016/j.immuni.2012.08.016 PMC3525757 23123063

[27] Zohar Y Wildbaum G Novak R Salzman AL Thelen M Alon R. CXCL11-dependent induction of FOXP3-negative regulatory T cells suppresses autoimmune encephalomyelitis. J Clin Invest. (2014) 124:2009–22. doi: 10.1172/JCI71951 PMC4001543 24713654

[28] Karin N. Chemokines in the landscape of cancer immunotherapy: how they and their receptors can be used to turn cold tumors into hot ones? Cancers (Basel). (2021) 13:6317. doi: 10.3390/cancers13246317 34944943 PMC8699256

[29] Karin N. CXCR3 ligands in cancer and autoimmunity, chemoattraction of effector T cells, and beyond. Front Immunol. (2020) 11:976. doi: 10.3389/fimmu.2020.00976 32547545 PMC7274023

[30] Karin N. Chemokines and cancer: new immune checkpoints for cancer therapy. Curr Opin Immunol. (2018) 51:140–5. doi: 10.1016/j.coi.2018.03.004 29579623

[31] Luster AD Unkeless JC Ravetch JV. Gamma-interferon transcriptionally regulates an early-response gene containing homology to platelet proteins. Nature. (1985) 315:672–6. doi: 10.1038/315672a0 3925348

[32] Luster AD Ravetch JV. Biochemical cherecterization of gamma interferon inducible cytokine (IP-10). J Exp Med. (1987) 166:1084–97. doi: 10.1084/jem.166.4.1084 PMC2188708 2443596

[33] Kato M Takahashi M Akhand AA Liu W Dai Y Shimizu S. Transgenic mouse model for skin Malignant melanoma. Oncogene. (1998) 17:1885–8. doi: 10.1038/sj.onc.1202077 9778055

[34] Umansky V Sevko A. Ret transgenic mouse model of spontaneous skin melanoma: focus on regulatory T cells. Pigment Cell melanoma Res. (2013) 26:457–63. doi: 10.1111/pcmr.2013.26.issue-4 23560814

[35] Doron H Amer M Ershaid N Blazquez R Shani O Lahav TG. Inflammatory activation of astrocytes facilitates melanoma brain tropism via the CXCL10-CXCR3 signaling axis. Cell Rep. (2019) 28:1785–1798.e6. doi: 10.1016/j.celrep.2019.07.033 31412247

[36] Sierro F Biben C Martinez-Munoz L Mellado M Ransohoff RM Li M. Disrupted cardiac development but normal hematopoiesis in mice deficient in the second CXCL12/SDF-1 receptor, CXCR7. Proc Natl Acad Sci USA. (2007) 104:14759–64. doi: 10.1073/pnas.0702229104 PMC1976222 17804806

[37] Dalit L Alvarado C Kuijper L Kueh AJ Weir A D’Amico A. CXCL11 expressing C57BL/6 mice have intact adaptive immune responses to viral infection. Immunol Cell Biol. (2022) 100:312–22. doi: 10.1111/imcb.v100.5 PMC9542850 35233830

[38] Yu Y Cho HI Wang D Kaosaard K Anasetti C Celis E. Adoptive transfer of Tc1 or Tc17 cells elicits antitumor immunity against established melanoma through distinct mechanisms. J Immunol. (2013) 190:1873–81. doi: 10.4049/jimmunol.1201989 PMC3563723 23315072

[39] Imaizumi K Suzuki T Kojima M Shimomura M Sakuyama N Tsukada Y. Ki67 expression and localization of T cells after neoadjuvant therapies as reliable predictive markers in rectal cancer. Cancer Sci. (2020) 111:23–35. doi: 10.1111/cas.v111.1 31660687 PMC6942445

[40] Classe M Burgess A El Zein S Wassef M Herman P Mortuaire G. Evaluating the prognostic potential of the Ki67 proliferation index and tumour-infiltrating lymphocytes in olfactory neuroblastoma. Histopathology. (2019) 75:853–64. doi: 10.1111/his.13954 31306501

[41] Friedrich J Seidel C Ebner R Kunz-Schughart LA. Spheroid-based drug screen: considerations and practical approach. Nat Protoc. (2009) 4:309–24. doi: 10.1038/nprot.2008.226 19214182

[42] Barash U Zohar Y Wildbaum G Beider K Nagler A Karin N. Heparanase enhances myeloma progression via CXCL10 downregulation. Leukemia. (2014) 28:2178–87. doi: 10.1038/leu.2014.121 PMC4185261 24699306

[43] Kuchroo VK Anderson AC Petrovas C. Coinhibitory receptors and CD8 T cell exhaustion in chronic infections. Curr Opin HIV AIDS. (2014) 9:439–45. doi: 10.1097/COH.0000000000000088 25010894

[44] Blackburn SD Shin H Haining WN Zou T Workman CJ Polley A. Coregulation of CD8+ T cell exhaustion by multiple inhibitory receptors during chronic viral infection. Nat Immunol. (2009) 10:29–37. doi: 10.1038/ni.1679 19043418 PMC2605166

[45] Wherry EJ Kurachi M. Molecular and cellular insights into T cell exhaustion. Nat Rev Immunol. (2015) 15:486–99. doi: 10.1038/nri3862 PMC4889009 26205583

[46] Sato Y Fu Y Liu H Lee MY Shaw MH. Tumor-immune profiling of CT-26 and Colon 26 syngeneic mouse models reveals mechanism of anti-PD-1 response. BMC Cancer. (2021) 21:1222. doi: 10.1186/s12885-021-08974-3 34774008 PMC8590766

[47] Ware MB Phillips M McQuinn C Zaidi MY Knochelmann HM Greene E. Dual IL-6 and CTLA-4 blockade regresses pancreatic tumors in a T cell- and CXCR3-dependent manner. JCI Insight. (2023) 8:1–16. doi: 10.1172/jci.insight.155006 PMC10243806 36881480

[48] House IG Savas P Lai J Chen AXY Oliver AJ Teo ZL. Macrophage-derived CXCL9 and CXCL10 are required for antitumor immune responses following immune checkpoint blockade. Clin Cancer Res. (2020) 26:487–504. doi: 10.1158/1078-0432.CCR-19-1868 31636098

[49] Hashimoto M Araki K Cardenas MA Li P Jadhav RR Kissick HT. PD-1 combination therapy with IL-2 modifies CD8(+) T cell exhaustion program. Nature. (2022) 610:173–81. doi: 10.1038/s41586-022-05257-0 PMC9793890 36171288

[50] Li H Leun AMvd Yofe I Lubling Y Gelbard-Solodkin D Akkooi ACJv. Dysfunctional CD8 T cells form a proliferative, dynamically regulated compartment within human melanoma. Cell. (2019) 176:775–789.e18. doi: 10.1016/j.cell.2018.11.043 30595452 PMC7253294

[51] Kamphorst AO Wieland A Nasti T Yang S Zhang R Barber DL. Rescue of exhausted CD8 T cells by PD-1-targeted therapies is CD28-dependent. Science. (2017) 355:1423–7. doi: 10.1126/science.aaf0683 PMC5595217 28280249

[52] Yokota E Iwai M Yukawa T Naomoto Y Haisa M Monobe Y. Patient-derived tumoroid models of pulmonary large-cell neuroendocrine carcinoma: a promising tool for personalized medicine and developing novel therapeutic strategies. Cancer Lett. (2024) 588:216816. doi: 10.1016/j.canlet.2024.216816 38499265

[53] Zhang Z Chen X Gao S Fang X Ren S. 3D bioprinted tumor model: a prompt and convenient platform for overcoming immunotherapy resistance by recapitulating the tumor microenvironment. Cell Oncol (Dordr). (2024) 47:1113–26. doi: 10.1007/s13402-024-00935-9 PMC11322267 38520648

[54] Mo F Yu Z Li P Oh J Spolski R Zhao L. An engineered IL-2 partial agonist promotes CD8(+) T cell stemness. Nature. (2021) 597:544–8. doi: 10.1038/s41586-021-03861-0 PMC9172917 34526724

[55] Oh DY Kwek SS Raju SS Li T McCarthy E Chow E. Intratumoral CD4(+) T cells mediate anti-tumor cytotoxicity in human bladder cancer. Cell. (2020) 181:1612–1625.e13. doi: 10.1016/j.cell.2020.05.017 32497499 PMC7321885

[56] Quezada SA Simpson TR Peggs KS Merghoub T Vider J Fan X. Tumor-reactive CD4(+) T cells develop cytotoxic activity and eradicate large established melanoma after transfer into lymphopenic hosts. J Exp Med. (2010) 207:637–50. doi: 10.1084/jem.20091918 PMC2839156 20156971

[57] Mikucki ME Fisher DT Matsuzaki J Skitzki JJ Gaulin NB Muhitch JB. Non-redundant requirement for CXCR3 signalling during tumoricidal T-cell trafficking across tumour vascular checkpoints. Nat Commun. (2015) 6:7458. doi: 10.1038/ncomms8458 26109379 PMC4605273

[58] Wen J Ye F Huang X Li S Yang L Xiao X. The tumor-to-breast volume ratio (TBR) predicts cancer-specific survival in breast cancer patients who underwent modified radical mastectomy. Tumour Biol. (2016) 37:7493–500. doi: 10.1007/s13277-015-4382-2 26678892

