# Supplementary figures and images for: PD-1 and CTLA-4 serve as major gatekeepers for effector and cytotoxic T-cell potentiation by limiting a CXCL9/10-CXCR3-IFNγ positive feedback loop
Source: Front Immunol. 2024 Oct 15;15:1452212. doi: 10.3389/fimmu.2024.1452212 (PMC11519525; doi:10.3389/fimmu.2024.1452212)

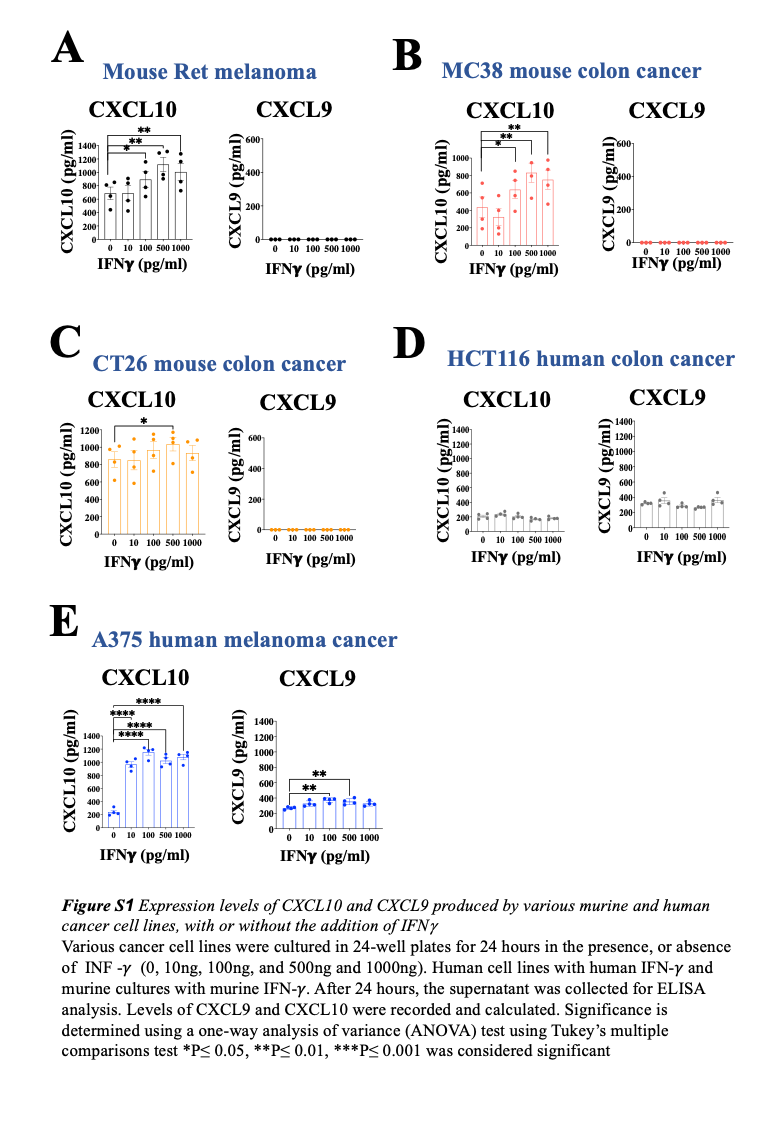

Supplement: Supplementary file 1 [file Image1.tiff]

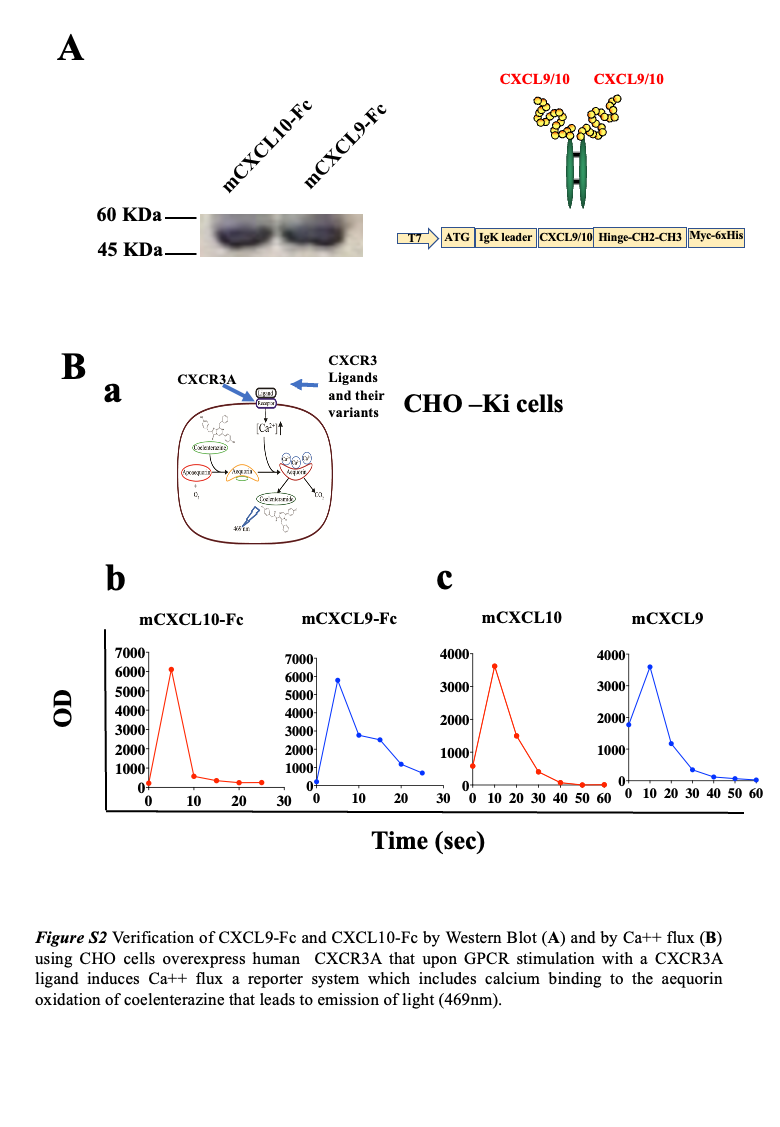

Supplement: Supplementary file 2 [file Image2.tiff]

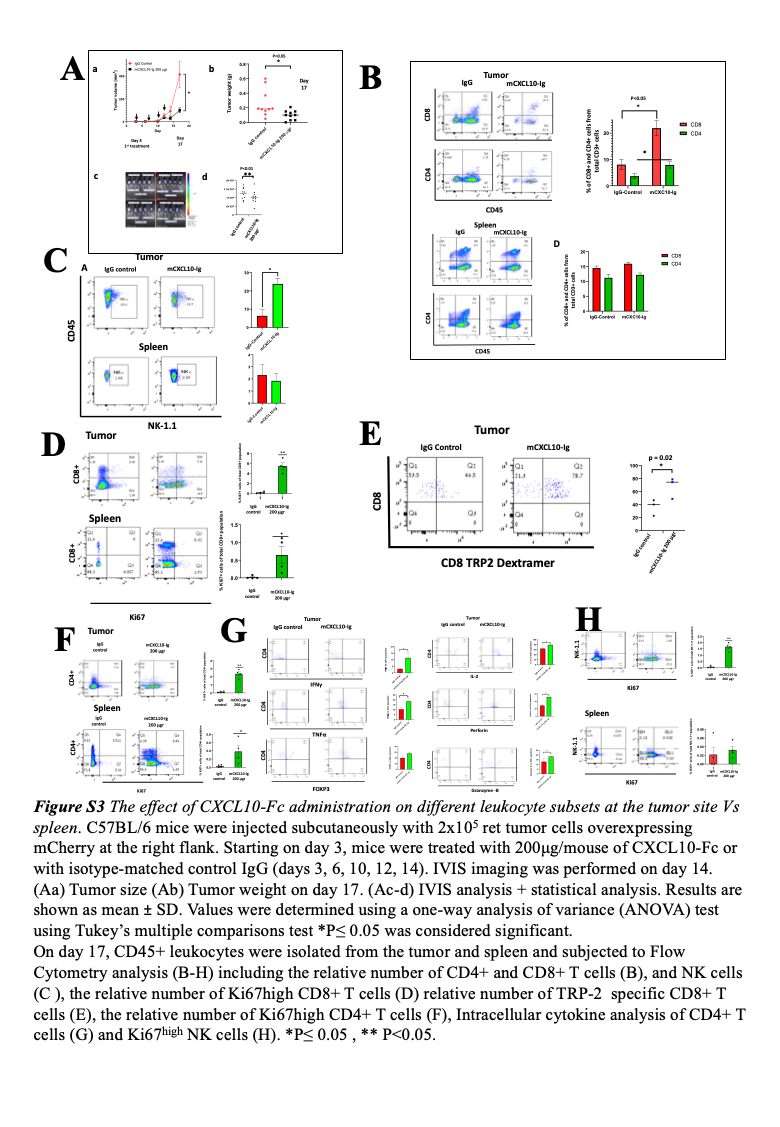

Supplement: Supplementary file 3 [file Image3.tiff]
